# Lipid rafts disruption by statins negatively impacts the interaction between SARS-CoV-2 S1 subunit and ACE2 in intestinal epithelial cells

**DOI:** 10.3389/fmicb.2023.1335458

**Published:** 2024-01-08

**Authors:** Marianne El Khoury, Hassan Y. Naim

**Affiliations:** Department of Biochemistry, University of Veterinary Medicine Hannover, Hannover, Germany

**Keywords:** lipid rafts, statins, protein–protein interaction, SARS-CoV-2, protein trafficking

## Abstract

The causative agent of the COVID-19 pandemic, SARS-CoV-2, is a virus that targets mainly the upper respiratory tract. However, it can affect other systems such as the gastrointestinal (GI) tract. Therapeutic strategies for this virus are still inconclusive and understanding its entry mechanism is important for finding effective treatments. Cholesterol is an important constituent in the structure of cellular membranes that plays a crucial role in a variety of cellular events. In addition, it is important for the infectivity and pathogenicity of several viruses. ACE2, the main receptor of SARS-CoV-2, is associated with lipid rafts which are microdomains composed of cholesterol and sphingolipids. In this study, we investigate the role of statins, lipid-lowering drugs, on the trafficking of ACE2 and the impact of cholesterol modulation on the interaction of this receptor with S1 in Caco-2 cells. The data show that fluvastatin and simvastatin reduce the expression of ACE2 to variable extents, impair its association with lipid rafts and sorting to the brush border membrane resulting in substantial reduction of its interaction with the S1 subunit of the spike protein. By virtue of the substantial effects of statins demonstrated in our study, these molecules, particularly fluvastatin, represent a promising therapeutic intervention that can be used off-label to treat SARS-CoV-2.

## 1 Introduction

The COVID-19 pandemic has been affecting individuals worldwide and causing many deaths since the discovery of its causative agent, the severe acute respiratory syndrome coronavirus 2 (SARS-CoV-2) in December 2019 (Huang et al., 2020). Although SARS-CoV-2 infects the host via the respiratory tract, other systems in the human body have also been affected (Zou et al., 2020). For instance, infected patients have reported gastrointestinal-related signs and symptoms such as discomfort, nausea, vomiting, and diarrhea (Wu et al., 2022). In addition to symptoms reported during infection, an increase in functional gastrointestinal disorders (FGID) was observed post-COVID, such irritable bowel syndrome (IBS) (Ghoshal et al., 2022; Marasco et al., 2022). A better understanding of the multisystem impact of SARS-CoV-2 would give more insights into its entry and infection mechanism, therefore facilitating the discovery of new therapies.

This virus gains entry by interacting mainly with angiotensin-converting enzyme 2 (ACE2), a type I transmembrane metalloprotease expressed at the surface of many cell types (Hikmet et al., 2020; Zhou et al., 2020). ACE2 is associated with lipid microdomains, also known as lipid rafts (LR) (Lu et al., 2008). They are mainly composed of cholesterol and sphingolipids and are essential for the trafficking and sorting of the ACE2 receptor, as well as other transmembrane proteins, to the cell surface (Simons and Ikonen, 1997; Lu et al., 2008). Several studies correlated LR with the interaction, entry, and stability of many viruses such as influenza, respiratory syncytial viruses, and SARS-CoV-1 (Glende et al., 2008; Lu et al., 2008; Bajimaya et al., 2017a), highlighting their suitability as therapeutic targets.

A major component of LR is cholesterol, a fundamental constituent in the structure of cellular membranes that plays a crucial role in a variety of cellular events such as signal transduction, immune function, and protein sorting to name a few (Pikuleva, 2006; McLean et al., 2012). The disruption of cholesterol synthesis has been demonstrated to impact the infectivity and pathogenicity of several viruses (Glende et al., 2008; Bajimaya et al., 2017b). In fact, SARS-CoV-2 infection was also found to be LR dependent as demonstrated by cholesterol depletion with cyclodextrins such as methyl-β-cyclodextrin and alpha-cyclodextrin (Ergoren et al., 2020; Li et al., 2021; Raïch-Regué et al., 2023).

Cholesterol synthesis can be blocked by a group of molecules collectively known as statins, which target HMG-CoA reductase, an enzyme crucial for cholesterol biosynthesis (Endo, 1992). This inhibitory capacity of statins may have, in turn, implications on the integrity of LR and render these molecules attractive drugs in affecting the interaction of viruses with those receptors that are associated with LR.

Statins are approved molecules that are prescribed for several treatments, such as cancer, hyperlipidemia, or for the prevention of cardiovascular diseases (Zhou and Liao, 2009; Morofuji et al., 2022). Repurposing these drugs constitute a safe and fast track to finding suitable therapies against SARS-CoV-2 given that treatments are still needed. Many studies have elucidated the promising effect of statins on SARS-CoV-2 (Sorice et al., 2020; Zapatero-Belinchón et al., 2021; Teixeira et al., 2022). However, more studies are required to further understand the mechanism underlying the effect exerted by these drugs, not only on lung cells, but also on other cell types that are affected by SARS-CoV-2. Investigating the effects exerted by statins on the different organs affected by the virus would give further information on the impact and suitability of these approved drugs as antiviral agents.

In this study, we used fluvastatin and simvastatin to investigate the effect of cholesterol inhibition on the interaction between the S1 subunit of SARS-CoV-2 spike protein, and ACE2 at the surface of intestinal epithelial cells. We show that both statins negatively impact the trafficking of ACE2 and impair its sorting to the intestinal brush border or apical membrane with the consequence that the interaction of S1 with ACE2 is significantly reduced.

## 2 Materials and methods

### 2.1 Cell culture

COS-1 (American Type Culture Collection, Mannasas, VA, United States), and Caco-2 cells (DSMZ, Braunschweig, Germany) were maintained in Dulbecco’s Modified Eagle Medium (DMEM) 1 g/L glucose and DMEM 4.5 g/L glucose, respectively (ThermoFisher Scientific, Waltham, MA, United States), supplemented with 10% fetal calf serum (FCS) and penicillin–streptomycin (Sigma-Aldrich, Darmstadt, Germany). Both cell lines were passaged and maintained in a 5% CO_2_ humidified incubator at 37°C.

### 2.2 Transient transfection and pulldown experiments

COS-1 cells were transiently transfected using the DEAE-Dextran method. Briefly, the cells were seeded the day before transfection. DEAE-Dextran was incubated with 3 μg of cDNA encoding the S1 subunit fused to Fc from human IgG (pCG1_sol-SARS-2-S1-Fc), kindly provided by Dr. Markus Hoffmann (Infection Biology Unit, German Primate Center, Göttingen, Germany) in transfection medium (DMEM 1 g/L) and added to COS-1 cells. The DEAE-Dextran/DNA complex was removed after 90 min and complete media containing chloroquine was added for 4 h. Chloroquine was then removed and replaced with complete media. The following day, media without FCS was added over the transfected COS-1 cells. Media was collected 48 h post-transfection for further analysis and cells were lysed with 1% Triton X-100 in 10 mM Tris, 150 mM NaCl, pH 7.4 (TX-100 lysis buffer) for 2 h at 4°C. Protein A-Sepharose® beads were added to the lysates to capture S1 proteins followed by immunoblotting.

### 2.3 Treatments with statins and protein extraction

Fluvastatin and simvastatin purchased from Sigma-Aldrich (Darmstadt, Germany), were dissolved in water and DMSO, respectively. Caco-2 cells were treated with 100 μM of either statin for 48 h. Treated, and non-treated cells were similarly viable and showed no changes by light microscopy. The cells were then washed twice with ice-cold PBS (pH 7.4) and lysed with TX-100 lysis buffer for 2 h at 4°C. The samples were centrifuged at 17,000 × g for 20 min at 4°C and protein concentration was measured using Bradford Assay. An equal amount of proteins was subjected to SDS-PAGE followed by immunoblotting using antibodies described in Table 1.

**Table 1:** Primary and secondary antibodies.

Antibody	Concentration	Dilution	Company/Reference	Cat #
Recombinant anti-ACE2 antibody	0.231 μg/μL	1:5,000	Abcam	ab108252
Flotillin-2 (B-6)	0.2 μg/μL	1:5,000	Santa Cruz Biotechnology	sc-28320
Protein A (HRP Conjugate)	-	1:1,000	Cell Signaling	12291
Actin	2 μg/μL	1:5,000	Santa Cruz Biotechnology	sc-47778
mAb HBB 3/775/42 (DPPIV)	-	1:5,000	Hauri et al. (1985)	-
HSI 9 (SI)	-	1:2,500	Beaulieu et al. (1989)	-
Goat anti-mouse IgG (H + L) Secondary antibody HRP	0.4 μg/μL	1:5,000	ThermoFisher Scientific	31430
Goat anti-rabbit IgG HRP	0.4 μg/μL	1:5,000	ThermoFisher Scientific	31460

### 2.4 Brush border membrane isolation from Caco-2 cells

Caco-2 cells were treated as mentioned previously. Brush border membranes were prepared by the divalent CaCl_2_ procedure essentially as described by Schmitz et al. (1973) and the modification by Sterchi and Woodley (1980). In brief, the cells were washed twice with ice-cold PBS and homogenized in 2 mM Tris, 50 mM Mannitol, pH 7.0 (homogenization buffer). The homogenates (H) were subjected to 2 consecutive freezing and thawing cycles and sonication followed by centrifugation at 5,000 × g for 15 min at 4°C to remove debris. CaCl_2_ was added to the supernatant and left rotating for 30 min at 4°C followed by centrifugation at 5,000 × g for 15 min at 4°C to isolate the intracellular and basolateral membrane (P1, IM/BLM). The supernatant containing the apical membrane and cytosolic vesicles was subjected to ultracentrifugation at 25,000 × g for 30 min at 4°C and the pellet P1 was resuspended in the homogenization buffer. After ultracentrifugation, the supernatant containing the cytosolic vesicles (S) was collected and the apical membrane (P2) was resuspended in the homogenization buffer. The protein concentration of each fraction was measured using Bradford assay and an equal amount of protein was loaded on SDS-PAGE followed by immunoblotting.

### 2.5 Lipid raft isolation from Caco-2 cells

Control and treated Caco-2 cells were washed twice with ice-cold PBS and lysed with 1% Lubrol in PBS (pH 7.4). Lipid raft (LR) isolation was then performed according to the method by Shimada et al. (2005) and the modifications described by Wanes et al. (2021). Briefly, the lysates were homogenized 20 times through a 20G needle and kept for 2 h at 4°C. The samples were then centrifuged at 5,000 × g for 15 min at 4°C. The samples were loaded on a discontinuous sucrose gradient and subjected to ultracentrifugation at 100,000 × g for 18 h at 4°C. The gradients were divided into lipid raft (LR) and non-lipid raft (NLR) fractions collected from top to bottom. An equal volume of each fraction was mixed with Laemmli buffer and subjected to SDS-PAGE followed by immunoblotting.

### 2.6 Lipid extraction from LR and analysis

Total lipids were extracted from LR fractions obtained from control and treated Caco-2 cells (see previous section for LR isolation), using the method described by Brogden et al. (2014) with minor modifications. Cholesterol levels were analyzed using a Hitachi Chromaster High-Performance Liquid Chromatograph (HPLC), as previously described in Branitzki-Heinemann et al. (2016) and Brogden et al. (2017).

### 2.7 S1 binding to ACE2

Caco-2 cells were treated as mentioned previously. After 48 h of treatment, the cells were washed twice with ice-cold PBS and media from transfected COS-1 cells were added. The secreted S1 proteins contained in the media were allowed to bind to ACE2 at the surface of Caco-2 for 2 h at 4°C. Cells were then lysed with TX-100 lysis buffer and Protein A-Sepharose® beads were added to capture S1 proteins. The beads were then washed twice with TX-100 lysis buffer and subjected to SDS-PAGE followed by immunoblotting using anti-ACE2 antibodies.

### 2.8 SDS-PAGE and immunoblotting

SDS-PAGE Laemmli buffer containing DTT was added to the lysate or immunoprecipitates. The samples were boiled at 95°C for 5 min, resolved on 7% acrylamide gels, and transferred onto PVDF membranes. The membranes were then blocked in 5% skimmed milk before adding the respective primary antibodies (Table 1). The membranes were washed with PBS + 0.1% Tween20 and the corresponding secondary antibody was added (Table 1). After washing with the same buffer, the chemiluminescence signal was detected using ChemiDoc MP™ Touch Imaging System (Bio-Rad, Munich, Germany).

### 2.9 Statistical analysis

The experiments in this study were performed at least three times. The results are represented as mean ± standard error of the mean (SEM). Fluvastatin and simvastatin treated groups were compared to the DMSO control group. For statistical analysis, one- or two-way ANOVA followed by Tukey’s or Dunnett’s multiple comparison test were done using Graphpad Prism 9.0.0 (121; GraphPad Software, San Diego, CA, United States) and significance was shown as follows: ^*^p < 0.05, ^**^p < 0.01, ^***^p < 0.001, ^****^p < 0.0001.

## 3 Results

### 3.1 Fluvastatin and simvastatin reduce total ACE2 expression in Caco-2 cells

**Figure 1:**
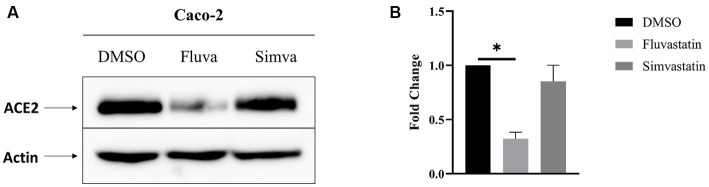
Fluvastatin and simvastatin reduce total ACE2 expression in Caco-2 cells. **(A)** Caco-2 cells were treated with fluvastatin or simvastatin for 48 h. The cells were lysed, and an equal amount of proteins was analyzed by immunoblotting using anti-ACE2 antibodies. β-actin was used as a loading control. **(B)** The results obtained in A were normalized to the internal control β-actin. Dunnett’s multiple comparisons test, ^*^p < 0.05, vs. DMSO, S.E.M., *n* = 3.

The effect of statins on the expression of ACE2 in intestinal epithelial Caco-2 cells was assessed in the presence or absence of cholesterol inhibitors, namely fluvastatin and simvastatin. Caco-2 cells were treated with these statins for 48 h and the expression of ACE2 assessed by Western blotting. Figure 1 shows that total ACE2 expression was markedly reduced by 68% upon treatment with fluvastatin, while it was reduced by only 15% after treatment with simvastatin, suggesting that despite the common mode of action of both drugs, other factors such as absorption, half-life and metabolic rate could lead to varying outcomes.

### 3.2 Fluvastatin and simvastatin affect the sorting of ACE2, DPPIV, and SI to the brush border membrane of Caco-2 cells

**Figure 2:**
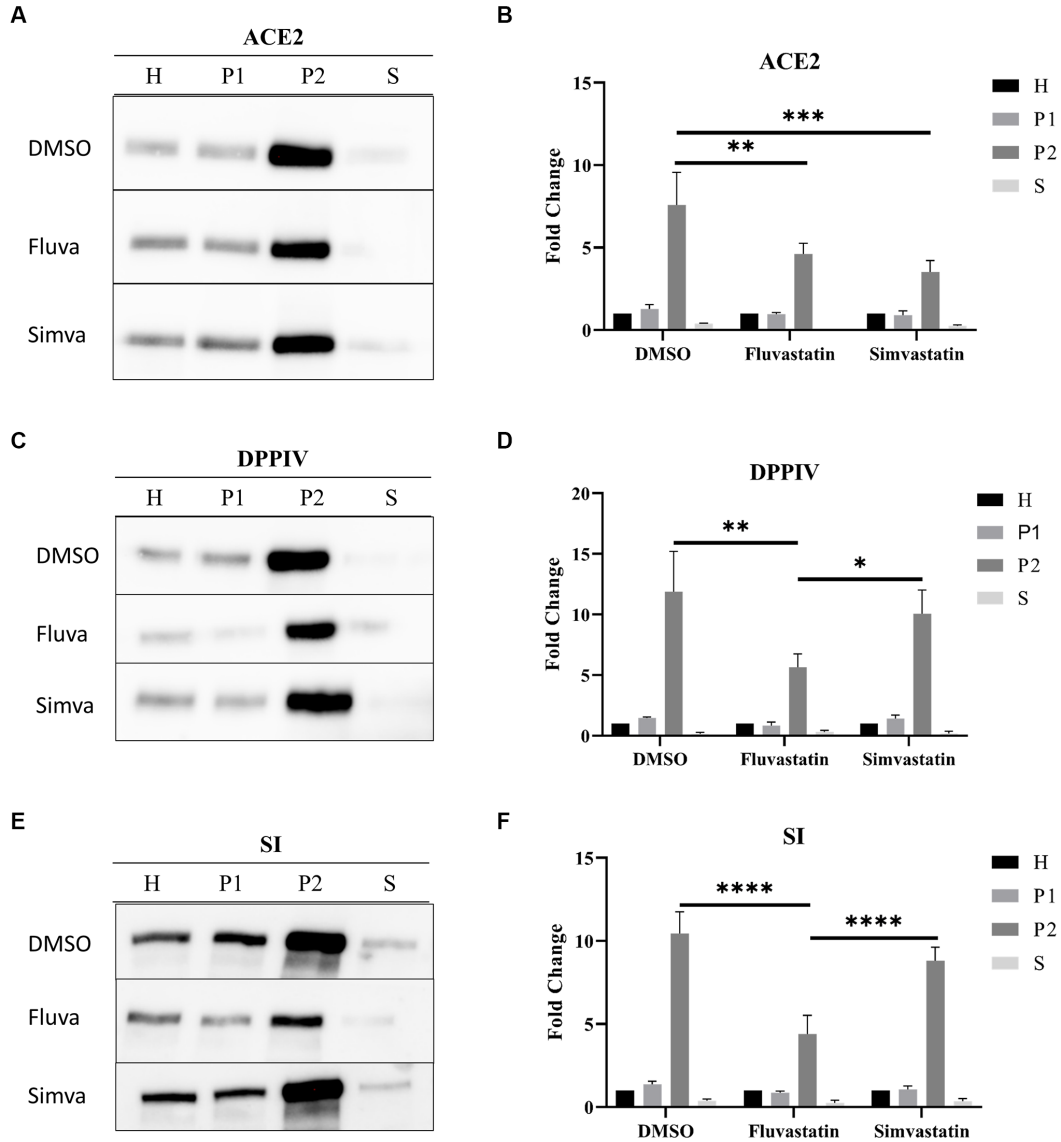
Fluvastatin and simvastatin affect the sorting of ACE2, DPPIV, and SI to the brush border membrane of Caco-2 cells. **(A)** Control and treated Caco-2 cells were homogenized 48 h post-treatment. The homogenates were then fractionated into H, P1, P2, and S using the divalent cation (CaCl_2_) method. Equal amounts of proteins from each fraction were analyzed by immunoblotting with anti-ACE2 antibodies. **(B)** Results obtained in A were normalized to the respective H, which was used as an internal control. The same procedure as described in A except that **(C,D)** anti-DPPIV antibodies and **(E,F)** anti-SI antibodies were used. Tukey’s multiple comparisons test, ^*^p < 0.05, ^**^p < 0.01, ^***^p < 0.001, ^****^p < 0.0001, vs. DMSO P2, S.E.M., *n* = 3.

ACE2 is mainly found in the apical or brush border membrane of polarized cells such as Caco-2. To evaluate the effect of statins on the trafficking of ACE2 to the apical or brush border membrane (BBM), the cells were treated as previously mentioned. The cellular homogenates (H) were fractionated into brush border membranes (BBM; P2 fraction), intracellular and basolateral membranes (IM/BLM; P1 fraction), and cytosolic and vesicular fraction (S). The proportions of ACE2 in P2 were assessed vs. that in H. Figures 2A,B show that both statins have substantially impaired the trafficking and sorting of ACE2 to BBM as assessed by the reduction of ACE2 in P2 of fluvastatin- and simvastatin-treated Caco-2 cells to 4.61 ± 0.65 and 3.52 ± 1.20, respectively, as compared to 7.60 ± 1.97 folds in P2 of the non-treated control cells.

We further studied the trafficking of other glycoproteins, dipeptidyl peptidase IV (DPPIV), a transmembrane protein involved in glucose homeostasis, and sucrase isomaltase (SI), a major disaccharidase located in the BBM and involved in carbohydrate digestion. Figures 2C,D show a marked impairment in the trafficking of DPPIV to BBM as assessed by the reduction of the proportion of DPPIV in P2 from 11.89 ± 3.32 folds to 5.65 ± 1.11 folds after treatment of Caco-2 cells with fluvastatin, while its trafficking was slightly reduced to 10.06 ± 1.96 folds following simvastatin treatment (Figures 2C,D). Similar results were also obtained for SI (Figures 2E,F). Notably, ACE2, DPPIV and SI are membrane glycoproteins that are sorted with high fidelity to the apical membrane via LR as platforms. Therefore, cholesterol inhibition by statins, particularly fluvastatin, could have resulted in LR alteration resulting thus in impaired sorting of these proteins to BBM.

### 3.3 Fluvastatin and simvastatin disrupt the association of proteins with lipid rafts

**Figure 3:**
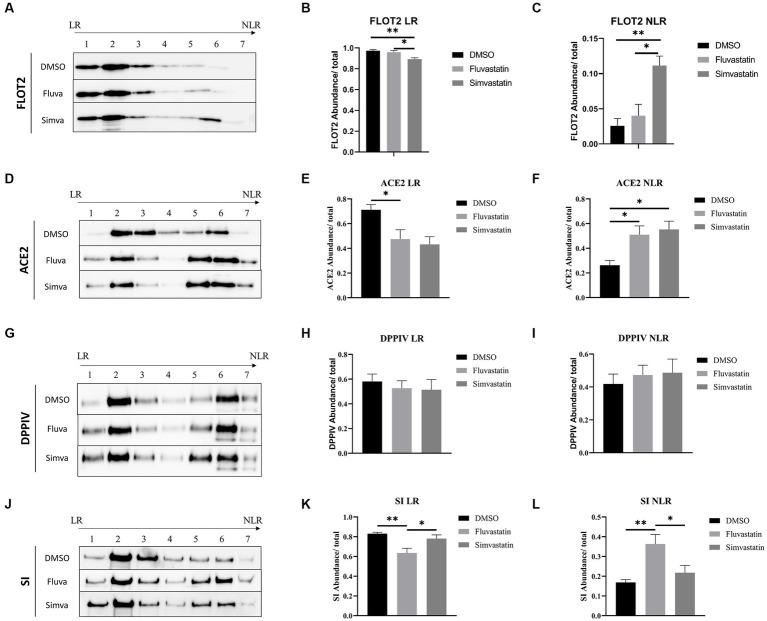
Fluvastatin and simvastatin disrupt the association of proteins with lipid rafts (LR). **(A)** Caco-2 cells were treated with DMSO, fluvastatin, or simvastatin for 48 h. The cells were homogenized, lysed with 1% Lubrol in PBS, and subjected to a discontinuous sucrose gradient. The fractions were then collected, and an equal volume was analyzed by immunoblotting with anti-flottilin-2 antibodies. **(B)** LR and **(C)** NLR were normalized to the total fractions. The same procedure described in A, except that **(D–F)** anti-ACE2 antibodies, **(G–I)** anti-DPPIV antibodies, and **(J–L)** anti-SI antibodies were used. Tukey’s multiple comparisons test, ^*^p < 0.05, ^**^p < 0.01, vs. DMSO, S.E.M., *n* = 5.

As indicated above, the marked effects of statins on the sorting of ACE2, DPPIV, and SI suggest that LR are distorted. LR are mainly composed of sphingolipids and cholesterol and are involved in protein sorting to the apical membrane of polarized cells. To evaluate the effects of fluvastatin and simvastatin on the association of the previously mentioned proteins with LR, control and treated samples were fractionated by discontinuous sucrose gradients and ultracentrifugation. Due to their low buoyant density, LR float into the top fractions of the gradient, while NLR are found in the last 3 fractions. Flotillin-2 (FLOT2) was used as a LR marker. Upon treatment of Caco-2 with fluvastatin and simvastatin, reduced levels of FLOT2 in the upper fractions were detected concomitant with reduced levels of LR, while increased levels of FLOT2 in NLR were observed, with simvastatin exerting a greater effect than fluvastatin (Figures 3A–C). Similar results were observed with ACE2 with no significant difference between the two cholesterol inhibitors (Figures 3D–F). Conversely, upon treatment of Caco-2 with both statins, DPPIV association with LR was not significantly affected (Figures 3G–I). A decreased association of SI with LR was also observed in the presence of fluvastatin, while only a slight variation was observed upon treatment with simvastatin (Figures 3J–L).

Moreover, ACE2 as well as other LR-associated proteins were retained in the upper floating fraction 2 of the gradient rather than the top fraction compatible with altered composition of the LR due to statins function in reducing cholesterol concentration (Supplementary Figure 1). Total lipids were extracted from raft fractions of control and treated cells to further investigate the role of statins. The results show that cholesterol levels were reduced in the presence of fluvastatin as compared to simvastatin (Supplementary Figure 2). Altogether, the results unequivocally show a partial distortion of LR and reduced association of the membrane proteins ACE2, DPPIV, and SI with these altered LR. The consequence is an impaired final step along the secretory pathway of these proteins, which is the sorting to BBM.

### 3.4 Fluvastatin and simvastatin reduce the interaction of S1 with ACE2 at the cell surface of Caco-2 cells

**Figure 4:**
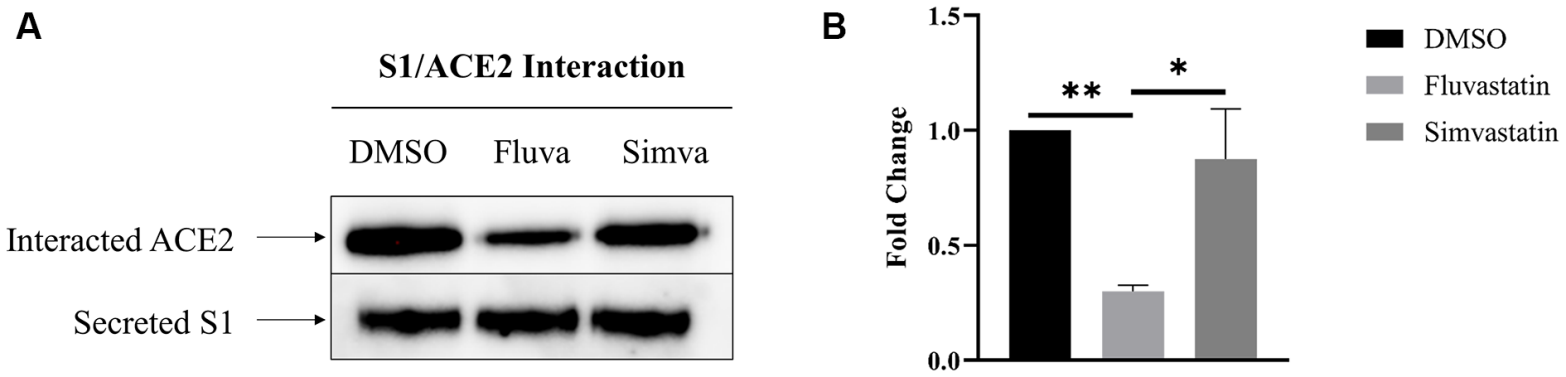
Fluvastatin and simvastatin reduce S1/ACE2 interaction at the cell surface of Caco-2 cells. **(A)** COS-1 cells were transiently transfected with the S1 subunit and Caco-2 cells were treated with fluvastatin or simvastatin for 48 h. Culture media containing the secreted S1 proteins was collected and added to Caco-2 cells for 2 h at 4°C to allow for S1/ACE2 binding. The cells were then lysed and S1 was captured using Protein A-Sepharose® beads. The samples were analyzed by immunoblotting using anti-ACE2 antibodies. **(B)** Results in A were analyzed and normalized to the control DMSO sample. Dunnett’s multiple comparisons test, ^*^p < 0.05, ^**^p < 0.01, vs. DMSO, S.E.M., *n* = 4.

To assess the effect of fluvastatin and simvastatin on the interaction between S1 and the ACE2 receptor at the cell surface of Caco-2 cells, culture media from transiently transfected COS-1 cells expressing S1 were added to Caco-2 cells at 4°C to avoid any potential endocytosis. The cells were then lysed, and Protein A-Sepharose® beads were used to pull-down S1 proteins that are tagged with the Fc-fragment of IgG. The samples were analyzed by immunoblotting using anti-ACE2 antibodies to detect potentially interacting ACE2. Upon treatment of Caco-2 with fluvastatin, almost 70% less interacting molecules were detected (Figure 4) compatible with the reduced levels of ACE2 in fluvastatin-treated Caco-2 cells (see above Figure 1A). The interaction of S1 with ACE2 produced in the presence of simvastatin was reduced by approximately 13%, which also reflects the slight reduction in the levels of ACE2 in the presence of this cholesterol inhibitor. The data show that interaction of ACE2 with S1 is reduced to different extents in the presence of the statins relative to reduced protein levels (Figure 1A) and expression in BBM (see Figure 2A). These findings suggest that the inhibition of cholesterol synthesis by statins could reduce SARS-CoV-2 infection.

## 4 Discussion

This study shows that treatment of intestinal epithelial cells with lipid-lowering agents, namely fluvastatin and simvastatin, distorted LR, reduced the expression of the SARS-CoV-2 receptor ACE2 to variable extents, and altered its association with LR and finally impaired its trafficking and sorting to the BBM. Strikingly and consequently, a substantial reduction in the amount of the ectodomain of the spike protein, S1, interacted with ACE2. Studies have demonstrated the association of ACE2 with lipid microdomains through colocalization with LR markers such as caveolin-1 and flottilin-2 (Glende et al., 2008; Lu et al., 2008). In a previous study with SARS-CoV-1 and ACE2, we could demonstrate that the depletion of membrane cholesterol by methyl-β-cyclodextrin results in changes in ACE2 localization in lipid microdomains and impairs infectivity of pseudo-SARS of Vero cells (Glende et al., 2008). In addition to its effect on ACE2 localization, methyl-β-cyclodextrin also affected ACE2 expression (Bakillah et al., 2022). The impact of cyclodextrins on SARS-CoV-2 has also been investigated and showed that these molecules affect the virus as well (Li et al., 2021; Raïch-Regué et al., 2023). The current results support the primordial role of cholesterol-rich LR in viral replication and the dependence of SARS-CoV-2 entry on LR. This is certainly in view of the complex structure of the microdomains in concentrating receptors and coreceptors, thereby affecting viral entry and subsequent steps in the infection process (Tang et al., 2019).

Statins are mainly prescribed for patients diagnosed with hypercholesterolemia and are efficient and safe (Schachter, 2005). Nonetheless, studies have shown that this family of drugs exerts pleiotropic effects, such as anti-inflammatory, anti-cancer, and immunomodulatory, to name a few (Oesterle et al., 2017; Ahmadi et al., 2020). Although all statins are competitive inhibitors for the same enzyme, they differ in their chemical structure, pharmacokinetic profile, and bioavailability. While fluvastatin is a hydrophilic drug with a half-life of less than an hour and 24% bioavailability, simvastatin is a lipophilic pro drug with a longer half-life of 3 h but less than 5% bioavailability. Moreover, simvastatin is converted into active metabolites (Chong et al., 2001). These differences could explain the various effects these statins exert on trafficking of ACE2, DPPIV, and SI. Furthermore, DPPIV was the least affected by statin treatments. This could be because it is indirectly sorted to the apical membrane via the transcytosis pathway, unlike SI and ACE2, which are sorted with high fidelity (Matter et al., 1990). The concentration of the statins used in this study is higher than the usual doses prescribed to patients (Zapatero-Belinchón et al., 2021). While our objective was to investigate the underlying mechanism of ACE2 trafficking and its interaction with S1, *in vivo* studies using appropriate concentrations are necessary.

Our data demonstrate impaired ACE2 trafficking to the brush border membrane of Caco-2 cells which resulted in lower interactions between S1 and ACE2. This reduced interaction stems also from an overall reduced levels of ACE2 in the statin-treated cells, whereby the reduction in the presence of fluvastatin is substantial. These lipid-lowering drugs activate the unfolded protein response (UPR) likely due to impaired prenylation of small GTPases triggering the accumulation of proteins in the ER, ER stress, and ultimately protein degradation (Mörck et al., 2009). In fact, simvastatin was shown to induce ER stress by the activation of ER stress markers such as IREα1 and BiP/GRP78 which in turn activate the UPR (Ghavami et al., 2012). Another potential explanation for the reduced intracellular levels of ACE2 could be an enhancement of the trafficking of ACE2 from endosomes to lysosomes in the presence of statins, mostly Fluvastatin, as has been shown for β-amyloid and amyloid precursor protein (Shinohara et al., 2010). This alteration in the endosomal-lysosomal pathway could be the results of isoprenoid-dependent changes in Rab proteins (Ostrowski et al., 2007; Shinohara et al., 2010). The combination of these two effects, reduced levels of ACE2 and impaired trafficking to BBM, altogether results in a substantial decrease in ACE2 levels at the apical membrane and subsequently low interacting complexes that comprise S1 and ACE2 in Caco-2 cells.

Clinical studies have documented the effects of statins on COVID-19 patients. For instance, a nationwide Swedish cohort study conducted by Santosa et al. showed that statins provide protective effects and lower mortality of COVID-19 patients (Santosa et al., 2022). In another clinical study conducted by Zhang et al., statins were found to reduce mortality among COVID-19 patients (Zhang et al., 2020). A longitudinal multicenter study revealed that statins were associated with lower cough and shortness of breath in long term diabetes mellitus patients (Sadeghdoust et al., 2023). Altogether, the use of statins could prove to be beneficial for the treatment of COVID-19 infections.

In summary, this study shows the marked effect of two statins, namely fluvastatin and simvastatin, on the synthesis and trafficking of ACE2 and ultimate reduction of the interacting S1 and ACE2 complexes at the cell surface of intestinal epithelial cells. These findings provide a better understanding of the mechanism behind the effects of statins and the clinical observations reported. The model used in this study partially reflects the *in vivo* situation. However, since S1 is only part of the spike protein, additional studies using live SARS-CoV-2 will be required. Further investigation of the impact of statins on the entry and fusion mechanism of the virus would give better insights for further consideration of lipid-lowering drugs for the treatment of COVID-19 patients.

## Data availability statement

The raw data supporting the conclusions of this article will be made available by the authors, without undue reservation.

## Ethics statement

Ethical approval was not required for the studies on humans in accordance with the local legislation and institutional requirements because only commercially available established cell lines were used.

## Author contributions

ME: Formal analysis, Validation, Visualization, Investigation, Methodology, Writing – original draft, Writing – review & editing. HN: Conceptualization, Formal analysis, Funding acquisition, Methodology, Project administration, Resources, Supervision, Validation, Visualization, Writing – review & editing.
