# Supplementary material for: Lipid rafts disruption by statins negatively impacts the interaction between SARS-CoV-2 S1 subunit and ACE2 in intestinal epithelial cells
Source: Front Microbiol. 2024 Jan 8;14:1335458. doi: 10.3389/fmicb.2023.1335458 (PMC10800905; doi:10.3389/fmicb.2023.1335458)
Supplement: Supplementary file 1 [file Data_Sheet_1.PDF]

Supplementary Material

1 Supplementary Figures

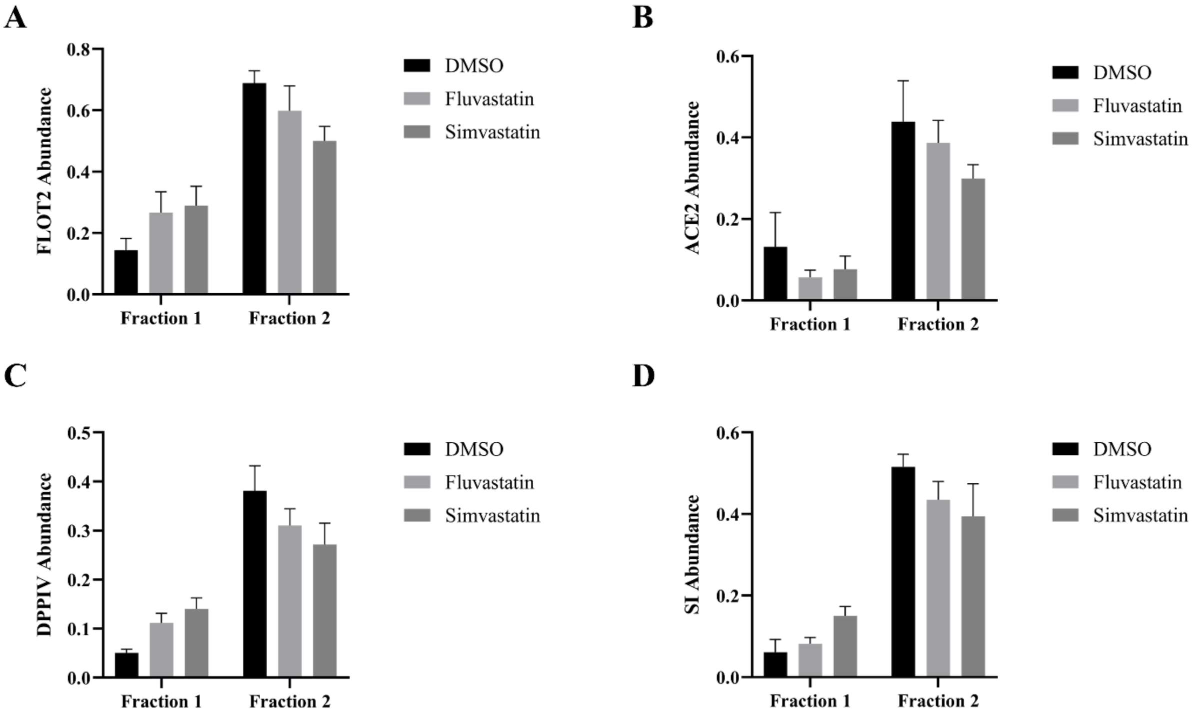

Supplementary Figure 1. Distribution of Proteins in Fractions 1 and 2 of Lipid Rafts. Caco-2 cells were treated with DMSO, fluvastatin or simvastatin for 48 hours. The cells were homogenized, lysed with 1% Lubrol in PBS, and subjected to a discontinuous sucrose gradient. **(A)** FLOT2, **(B)** ACE2, **(C)** DPPIV, and **(D)** SI. Šidák’s multiple comparison test, versus DMSO, S.E.M., $n = 4$.

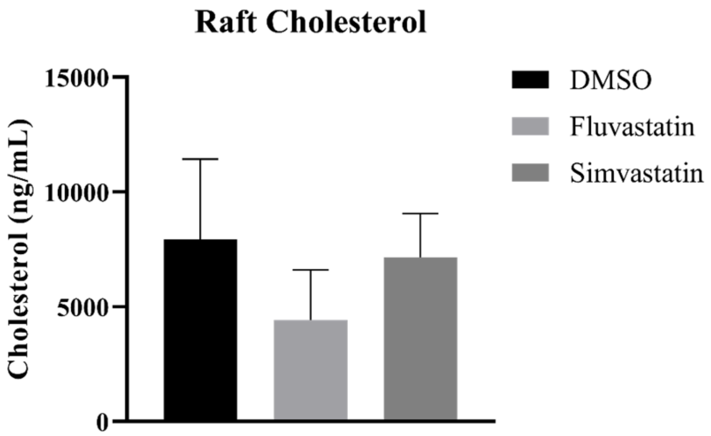

Supplementary Figure 2. Raft Cholesterol Levels. LRs were isolated from control and treated Caco-2 cells using a discontinuous sucrose gradient, and cholesterol was analyzed by HPLC. Tukey's multiple comparisons test, S.E.M., *n* = 3.
